# “Share Your Trauma… and Make It Funny”: Comedy as Catharsis and Community-Building in LGBTQ+ Mental Wellbeing and Resilience

**DOI:** 10.3390/bs16071059

**Published:** 2026-06-26

**Authors:** Anthony J. Gifford, Robert J. Beck, Daragh T. McDermott, Keeley Abbott

**Affiliations:** 1 Department of Psychology & Counselling, School of Life and Health Sciences, Birmingham City University, Birmingham B4 7BD, UK; keeley.abbott@bcu.ac.uk; 2 The Birmingham Repertory Theatre, Birmingham B1 2EP, UK; 3 School of Social Sciences, Nottingham Trent University, Nottingham NG1 4FQ, UK

**Keywords:** LGBTQ+ mental health, comedy, community, psychological resilience, arts-for-health

## Abstract

LGBTQ+ individuals experience disproportionately high rates of mental health challenges, driven largely by exposure to stigma, discrimination, and minority stress. In response, there is growing interest in creative, community-based interventions that support wellbeing. This study evaluates “LGBTQteehee!”, a 10-week stand-up comedy programme designed to promote mental wellbeing and resilience among LGBTQ+ individuals. *N* = 6 participants took part in post-intervention qualitative data collection via focus group and semi-structured interviews. Data were analysed using Reflexive Thematic Analysis. Two overarching themes were identified: “**Standing up Together: A Queer Ensemble**” and “**Comedy as Catharsis**”, each comprising two subthemes. The first theme captured the importance of community, highlighting “**Where Comedy Meets Community**” and “**Where Safety Takes Centre Stage**”, emphasising the role of belonging, diversity, and psychological safety. The second theme reflected the transformative potential of comedy, with “**Performing (Queer) Resilience**” and “**Taking the Mic and Owning the Moment**” illustrating how participants reframed lived experience into empowering narratives and reported increased confidence and self-expression. Findings suggest that the benefits of the intervention extend beyond humour alone, instead reflecting the interplay between social connection and identity-affirming narrative expression. Participants described how having a space to talk openly about their mental health and receive support from others contributed to their perceptions of both individual and collective resilience. These findings suggest that community-based comedy interventions may offer accessible and identity-affirming spaces through which LGBTQ+ individuals can explore wellbeing, belonging, and resilience. Whilst further evaluation is required, the findings contribute to growing discussions regarding the role of arts-based approaches in supporting LGBTQ+ mental health and wellbeing.

## 1. Introduction

Lesbian, gay, bisexual, transgender, queer and/or questioning (LGBTQ+) individuals face disproportionately high rates of mental health challenges (Nowaskie et al., 2024; Ream, 2022). Numerous studies document that LGBTQ+ people report higher levels of depression, anxiety, self-harm, and suicidal behaviour than the general population (McDonald, 2018; Plöderl & Tremblay, 2015). In a longitudinal analysis spanning 2010 to 2021, Bai et al. (2025) reported that LGBTQ+ individuals experienced greater increases in psychological distress compared with their heterosexual counterparts. Similarly, using data from a UK nationally representative cohort, Kim et al. (2024) found that sexual minority individuals, and in particular lesbian women, faced heightened mental health challenges during the COVID-19 pandemic.

These stark disparities are not solely due to one’s LGBTQ+ identity, but rather stem from the hostile social environments and stressors that many in the LGBTQ+ community endure (Matsick et al., 2024). Research shows that LGBTQ+ people frequently encounter discrimination, harassment, social rejection, and other stressors that can severely impact mental wellbeing (Kaprinis & Charalampakis, 2025). This pattern is explained by Minority Stress Theory, originally proposed by Meyer (2003), which posits that sexual and gender minorities experience unique and chronic stressors associated with their marginalised social status. These stressors include distal stressors, such as experiences of prejudice and discrimination, as well as proximal stressors, including expectations of rejection, concealment of identity, and internalised stigma. Consequently, societal factors drive these mental health inequalities, leaving sexual and gender minorities at greater risk of experiencing poorer mental health. Amid increasing hate crimes, regressive legislative agendas, and resurgent anti-LGBTQ+ discourse, these findings highlight the urgent need for co-ordinated, cross-sectoral interventions that address both the structural and psychosocial determinants of LGBTQ+ mental health (e.g., Robinson et al., 2025; Silva et al., 2025). Indeed, reflecting on two decades of scholarship, Frost and Meyer (2023) emphasise the continued relevance of Minority Stress Theory and argue that sustained efforts remain necessary to address the social conditions that continue to undermine LGBTQ+ wellbeing.

These pressing inequalities have prompted increasing exploration of alternative, creative modes of support (Fang et al., 2024). Among these, humour and comedy have emerged as distinctive art forms with the potential to ameliorate resilience, belonging, and emotional recovery (Jones et al., 2014). The truism that “laughter is the best medicine” has often been conceptualised through the notion of comic catharsis (Scheff & Scheeie, 2014). Originating in classical theories of theatre, catharsis broadly refers to the emotional processing and transformation that occurs through engagement with dramatic performance (e.g., Reckford, 1974; Westwood, 2004). Indeed, comedy may facilitate the processing of difficult emotional experiences by enabling audiences and performers to engage with adversity through humour and shared reflection (Turri, 2025). Importantly, comic catharsis is not limited to laughter alone, instead, comedy may evoke a range of emotional responses, including reflection, indignation, relief, and shared understanding, which can contribute to emotional regulation and psychological adaptation (Cohn, 2016).

Humour has long been recognised in psychology as a potential to buffer against adversity and notably, LGBTQ+ individuals often use humour as a coping strategy. For example, Craig et al. (2018) found that humour was a commonly reported coping strategy employed by queer youth, serving as a tool for bonding and resilience in the face of discrimination. However, humour-based coping is neither unique to LGBTQ+ populations nor uniformly beneficial, with its effects often dependent upon the context in which humour is used and experienced (Kruczek & Basińska, 2018). Yet, jokes shared within the community may enhance camaraderie and help individuals process painful experiences together, rather than in isolation (Craig et al., 2018). This aligns with broader findings that marginalised groups, such as the LGBTQ+ community, frequently develop rich traditions of comedic expression to navigate the hardships considering persistent discrimination (Borum & Chattoo, 2023). As such, the potential value of comedy-based interventions may lie not simply in humour itself, but in the structured, identity-affirming, and community-based contexts through which humour is collectively experienced and shared.

Beyond informal coping, there is emerging evidence that structured comedy activities (e.g., improvisational theatre or stand-up comedy) can positively impact mental health (Caslin & Davies, 2022). A systematic review of 17 comedy-based interventions demonstrated beneficial effects on mental health symptoms and psychosocial outcomes, including enhanced hope, identity, and empowerment (Kafle et al., 2023). Drawing on their dual role as a stand-up comic and academic, Barnes (2025) highlighted how stand-up comedy workshops offered a powerful medium for reframing traumatic experiences, cultivating social connectedness, and enhancing psychological and social resilience.

Resilience broadly refers to the capacity to adapt positively to adversity and maintain psychological functioning in the face of challenge (e.g., Breakwell, 2021). Through comedy, then, resilience may operate at multiple levels. At an individual level, the process of transforming difficult experiences into humorous narratives may facilitate emotional processing, cognitive reappraisal, and psychological resilience (Barnes, 2025). Yet socially, stand-up comedy is an inherently relational and dialogic art form (Brodie, 2014; Mintz, 1985); thus, it is plausible that belonging, mutual support, and collective resilience is enhanced through shared experiences and communal laughter. Moreover, comedy has long functioned as a vehicle for social critique and resistance, enabling marginalised groups to challenge dominant narratives, articulate alternative perspectives, and reclaim agency in the face of oppression (Quirk, 2015). Therefore, comedy as an art form is increasingly seen as a legitimate avenue for supporting mental wellbeing, through the enhancement of resilience. Early evaluations of such programmes (e.g., Kafle et al., 2023) report improvements in participants’ self-esteem and reduction in depressive and stress symptoms. This may be, in part, due to the comedy providing a platform of enjoyment, encouraging self-expression, and enabling people to articulate difficulties in a more light-hearted way.

Growing interest in arts-based health initiatives, including the use of comedy, reflects increasing evidence that creative engagement can boost individual and community wellbeing (Stickley & Hui, 2012). This development is unsurprising given the extensive literature demonstrating the importance of community belonging, social support, and social connectedness for LGBTQ+ mental health. Research consistently shows that connection to affirming communities can buffer the effects of minority stress and promote psychological wellbeing among sexual and gender minorities (e.g., Gifford & Jaspal, 2026; Hammack et al., 2024; Jaspal, 2025; Meyer, 2015). Importantly, these benefits are not unique to comedy-based interventions. Rather, comedy forms part of a growing body of scholarship recognising the arts as valuable mechanisms for community building and social support (Chappell et al., 2021; Fancourt, 2017; Stickley & Hui, 2012).

Specifically, however, comedy-based interventions might be especially beneficial for LGBTQ+ individuals by leveraging the “social cure” (Jetten et al., 2012). Drawing from Social Identity Theory approach to health (Haslam et al., 2009; Hornsey, 2008; Jetten et al., 2012; Tajfel, 1974; Tajfel et al., 2001), the social cure is an applied understanding that meaningful group based interventions can have psychosocial benefits and support mental wellbeing (e.g., McGlashan et al., 2025). The benefits to health and wellbeing are thought to arise through interactions between individual- and group-level mechanisms, including the reattribution of minority stress and the development of collective efficacy (Häusser et al., 2020). Social prescribing offers a pathway to put this into practice, connecting people—especially those at risk of marginalisation—with community activities like comedy workshops (Kafle et al., 2023). Considering long wait lists for mental health care, such interventions can provide more immediate and differential support for mental wellbeing and resilience (Punton et al., 2022). Despite promising evidence supporting comedy-based interventions, there remains a paucity of research examining their use within LGBTQ+ populations. Moreover, relatively little is known about how such interventions may support wellbeing, resilience, belonging, and community connectedness specifically among LGBTQ+ individuals.

Within this context, the present study focuses on a novel community arts-based initiative, “LGBTQteehee!”, a 10-week stand-up comedy workshop hosted at a theatre in Birmingham, UK. Developed in partnership with professional comedians and wellness practitioners, the programme used comedy as a vehicle for connection, empowerment, and the promotion of LGBTQ+ mental wellbeing and resilience. The free-to-access course invited LGBTQ+ individuals from across the West Midlands to participate in weekly sessions where they learned key elements of stand-up comedy, discovered their comedic voice, crafted an on-stage persona, and transformed lived experiences into empowering comedic material. The programme culminated in a five-minute live stand-up performance as part of a wider showcase celebrating mental health and the arts. All sessions took place within a safe and affirming group environment, underpinned by peer support and a sense of belonging among participants. A qualified counsellor was present throughout the programme and available for one-to-one support where required. Participants were introduced to principles of safe disclosure, retained full control over the personal material they shared, and were reminded that content could be modified, redacted, or withdrawn from the final showcase at any stage.

However, as Bungay et al. (2024) emphasise, for arts and health interventions to be effective, they must be subject to rigorous evaluation to ensure participant safety and to support the wider implementation of such programmes. This present study, therefore, was undertaken to understand participants’ experiences of “LGBTQteehee!” and how it may have supported their mental wellbeing and resilience. By qualitatively examining perspectives of this novel programme, this study seeks to assess whether this kind of arts-based approach is effective and feasible for larger scale or longer-term implementation. As such, this study takes a two-pronged approach. Firstly, it aims to contribute knowledge on whether comedy can be an effective medium for alleviating mental health burden among LGBTQ+ and other marginalised groups. Secondly, to understand participants’ experiences of “LGBTQteehee!” to enhance and develop iterative versions of the intervention. Specifically, the following two research questions are considered:

1. What are the experiences of participants engaging with the “LGBTQteehee!” intervention?
2. How do LGBTQ+ participants experience and describe the role of a stand-up comedy-based intervention in relation to their mental wellbeing?

## 2. Methods

### 2.1. Participants

A total of (*N* = 9) participants took part in the full 10-week programme comprising of three-hour weekly sessions, culminating in a five-minute solo stand-up comedy performance. The age range was 24–63 years, and all participants identified as part of the LGBTQ+ community, with (*n* = 3) identifying as lesbian, (*n* = 1) as gay, (*n* = 4) as bisexual, and (*n* = 1) as queer. Regarding gender identity, (*n* = 4) self-identified as cisgendered and (*n* = 5) as trans/gender diverse or non-binary. The majority of participants identified as White (*n* = 7), with (*n* = 1) participant identifying as South Asian and (*n* = 1) as Mixed Heritage. Table 1 highlights participant engagement in data collection and illustrates attrition following completion of the programme.

Participation in the follow-up data collection was optional, resulting in a final participant group of (*N* = 6) for the purposes of analysis. Although three participants did not participate in the follow-up data collection, they remained active members of the group throughout the intervention and successfully completed the programme, including the final public showcase.

### 2.2. Data Collection

Data collection took place after the intervention had finished between December 2024 and January 2025. Participants took part in a focus group, with the option to have an individual follow-up, one-to-one semi-structured interview. This approach balanced efficiency with data richness, while enabling participants to speak openly about their full experiences, which may have been constrained in a group setting (Lambert & Loiselle, 2008). Table 1 outlines key demographic characteristics and the chosen data collection modality; all names have been pseudonymised to protect participant confidentiality. Both the focus group and interviews were guided by open-ended questions, allowing space for the participants to freely discuss meaningful experiences relevant to them. However the interview schedule included guide topics such as: (1) participant experience, engagement, and group dynamics (e.g., How would you describe your overall experience of “LGBTQteehee!” from beginning to end?), (2) mental wellbeing, resilience, and queer identities (e.g., What stood out to you about the personal impact of participating in “LGBTQteehee!”?), and (3) perceived value, accessibility, and broader impact (e.g., How do you view “LGBTQteehee!” as an intervention for the wider LGBTQ+ community?). The focus group was scheduled for 120 min, and the interviews lasted between 45 and 75 min. All focus groups and interviews were conducted via Microsoft Teams, which generated automated transcripts. These transcripts were subsequently checked and edited verbatim by the research team.

### 2.3. Data Analysis

Reflexive Thematic Analysis (RTA; Braun & Clarke, 2021) was used to analyse the data. RTA is well suited to examining patterns of meaning across a corpus of qualitative data beyond a strictly idiographic focus, and it enables the concurrent analysis of data derived from both focus groups and individual interviews. While not a rigidly prescriptive approach, RTA involves an iterative, multi-stage analytic process:

1. As a collaborative analytic process, AG and KA immersed themselves in the dataset through repeated reading of the focus group transcript, ensuring shared familiarity with the core data.
2. An initial phase of coding was then undertaken, with all authors contributing to the coding of the focus group data. In addition, the lead author (AG) independently coded the one-to-one interview transcripts. Coding was informed by the research questions and an overarching theoretical framework and attended to both semantic content and underlying latent meanings.
3. AG and KA subsequently met to discuss the coding collaboratively, critically examining and refining codes to develop more nuanced and in-depth interpretations of the data. Insights from the one-to-one interview coding were used to supplement and enrich the analytic interpretations. DM provided supervisory oversight to mediate any disagreements.
4. Consistent with reflexive thematic analysis, analytic saturation was not treated as a fixed endpoint. Rather, recognising the inherently interpretive nature of qualitative analysis and the potential for ongoing meaning-making (Low, 2019), the research team made a situated and pragmatic judgement regarding when to move from coding to theme development (Braun & Clarke, 2021). At this stage, codes were considered sufficiently rich and refined to support the generation of themes that captured patterns of shared meaning across the dataset.
5. Initial themes were developed through the clustering and organisation of related codes and code labels.
6. These preliminary themes were then further developed and refined by the lead author, with supporting data extracts used to interrogate coherence, boundaries, and conceptual clarity.
7. The final themes were clearly defined and articulated, with illustrative participant quotations selected to support and evidence the analytic narrative.

### 2.4. Reflexivity

Reflexivity is widely recognised as a core component of rigorous qualitative research, requiring ongoing consideration of researcher positionality and its influence on the research process. The present study was situated within a critical realist perspective, which acknowledges the existence of an external reality whilst recognising that our understanding of that reality is inevitably mediated through social, cultural, and historical contexts (Braun & Clarke, 2023). It is also pertinent to acknowledge the positionality of the research team. All authors identify as White, queer and are cisgender (three men and one woman). These social locations inevitably shaped the lenses through which the data were interpreted.

Beyond salient identities, it is also important to acknowledge the power dynamics operating within both the intervention and research process. Two members of the research team (AG and RB) were directly involved in the design and delivery of the “LGBTQteehee!” programme. Furthermore, the lead author (AG) facilitated the programme and conducted the interviews. While this position likely contributed to the depth and richness of the data by facilitating rapport and trust with participants, it also introduced the potential for bias, particularly where there may be a tendency, conscious or otherwise, to privilege more positive accounts of the intervention.

To address this, the analytic process was conducted collaboratively. A second researcher (KA), who was not involved in the delivery of the programme, played a key role in coding and theme development, while DM provided supervisory oversight throughout the analytic process. It is also pertinent to acknowledge that AG, KA, and DM occupy different positions within the academic hierarchy. Although all were members of faculty, KA and DM held more senior positions than AG. These differing perspectives and institutional positions facilitated constructive challenge and critical discussion during analysis, creating opportunities to interrogate assumptions and minimise the influence of any single analytic perspective.

This collaborative process provided a degree of analytical distance while maintaining sensitivity to the experiences of LGBTQ+ participants and the wider aims of the intervention. Through this reflexive approach, we sought to balance insider knowledge and contextual understanding with critical scrutiny, thereby supporting a nuanced and credible interpretation of participants’ accounts.

## 3. Findings

A reflexive thematic analysis of the data generated two core themes, each comprising two sub-themes: (1) Standing up Together: A Queer Ensemble. This theme contains the following two subthemes: Where Comedy Meets Community and Where Safety Takes Centre Stage. The second core theme: (2) Comedy as Catharsis contains the following sub-themes: Performing (queer) Resilience and Taking the Mic and Owning the Moment. These themes capture the impact of the comedy intervention on participants’ wellbeing.

### 3.1. Standing Up Together: A Queer Ensemble

Participants’ accounts highlight the community building aspects of the intervention and how this facilitated the development of meaningful connections with those in the group, cultivating a sense of belonging. The empowering impact of community building on individuals was a recurring pattern throughout the data. Participants described how the process helped cultivate meaningful connections with group members. For many, the space provided a rare and valuable opportunity to meet and form relationships with other LGBTQ+ individuals, something that had been missing from their lives previously. The specific impact of building friendships and connections within the LGBTQteehee group played an important role in cultivating a sense of safety amongst group members.

### 3.2. Where Comedy Meets Community

For participants, the process of doing comedy as part of the intervention became an important vehicle for building connection and friendships. In discussing the benefits of the programme, many emphasised the friendships and community they were able to make through the process. This is evident in the following quote from Kelly:

*So the thing for me about like why the community element of it was like very important and impactful for me is [reference made to other member in the group] I don’t actually have a lot of queer friends that I haven’t made through like situations so like I do actually have loads of queer friends. But like it’s all like oh, but I know them through work and or I’ve made them through friends of friends like rather than, like, sort of getting out there and connecting with other people.*

*(Kelly)*

For Kelly, the perceived value of the intervention is closely tied to its community-based element. This centred on the opportunity to build connections with others in the LGBTQteehee group and being able to engage in meaningful and intentional interactions within queer spaces, facilitated by a shared purpose (comedy) rather than arising incidentally through other contexts or individuals.

While all participants emphasised the importance of community and friendships as some of the most impactful aspects of the process, for some, the opportunity to be part of a diverse and inclusive group held particular significance. Being part of the intervention offered them a unique opportunity to connect with individuals they might not ordinarily meet in their everyday lives.

*I think for me it was a sheer pleasure to get to know different people. So, people that did identify as gender fluid, people that did use different pronouns, trans people, I’ve had some friends from those areas, I’ve had some interactions, but not really as closely and it’s been a sheer pleasure just to be able to get involved with areas of the community that I perhaps wouldn’t have had that chance to otherwise.*

*(Nicola)*

*Diversity, coming together, you know everyone wanting to do stuff. I know that’s what we’re all saying. But that’s really what made it. That was the secret sauce.*

*(Nazeem)*

Nicola spoke specifically about valuing the opportunity to spend time with others who identify as gender diverse, highlighting the importance of fostering deeper connections through the group process. Here, connection through diversity, rather than shared identity, is emphasised. Nicola invokes this sense of community as something that emerges from the experience of diverse individuals coming together, through shared space and purpose. This is captured by Nazeem’s excerpt where he describes ‘*everyone wanting to do stuff*’. This phrase reflects a sense of collective motivation that unifies the group, creating connection through shared activity and engagement. In framing this as the ‘*secret sauce*’, Nazeem identifies it as the core ingredient that encapsulates the communal benefit of the intervention.

This sense of shared purpose and community afforded participants to right environment to practice and hone their comedy with support and encouragement.

*It’s every single one of those [reasons] throughout this entire thing, even at times when we might not have wanted to, all were constantly lifting each other up like we never would just went. That joke is truly awful. That’s it. Like or. You’re not funny. Why are you doing this? Like no one did that ever. And no one was there like. No one wants to hear about your trauma. Like none of that. It was just constant like. Oh, no. And if you change the whole wording, do this thing. And it was just like such a healthy, positive environment to be in.*

*(Kelly)*

For Kelly, the benefits of this community extended beyond receiving constructive and encouraging feedback on their work. Their impact was recognised most acutely in moments when the group offered emotional support, especially during times when members needed it more broadly. Having a positive group environment from which members could bond through their love of comedy, share stories and feedback, created a sense of community. This feeling of community was recognised by all participants and was fostered by the group’s shared sense of purpose and mutual appreciation of each other’s experiences.

### 3.3. Where Safety Takes Centre Stage

Participants described both the programme and the group as safe spaces. There was a strong recognition that psychological safety developed by the group, facilitators, and counsellor, was fundamental in helping them connect with the programme and openly share their experiences without fear of judgement. Participants collectively acknowledged this as unusual however. Many agreed that the facilitators were instrumental in establishing the right environment, particularly early in the programme before group dynamics had established.

*You guys [facilitators] obviously created the atmosphere and the safe space that relaxed us all enough to just open up and have an open heart and learn about new people.*

*(Athena)*

Here, Athena describes feeling a sense of safety, enabling them to express themselves more openly and in turn, become more receptive to others. Athena emphasises the importance and value of learning about others’ experiences and backgrounds, describing this as a transformative and beneficial aspect of the programme. She notes the crucial role the facilitators played in curating this environment, highlighting their supportive and reassuring approach. Shared identities were also significant in helping enable this process. Other participants also emphasised the significance of sharing space with those with similar backgrounds and identities, explaining how this allowed them to talk more openly about their mental health without having to worry about providing the usual justifications.

Participants described feeling a shared understanding amongst the group, as well as how this created a sense of validation and reprieve from the pressures to justify their struggles.

*As [Kelly] says, it can be so tiring out there in the world having to constantly explain or defend yourself, and actually sometimes I don’t need to, but just sometimes it’s that act of masking and pretending and pretending that you’re OK and pretending that everything’s fine and pretending that you’re normal when inside you just feel like you’re broken. And I’ve spent most of my life feeling like I’m broken. But when I’m with these guys, I don’t feel broken. I feel normal. And yeah, I feel that’s invaluable.*

*(Nicola)*

Here, Nicola reflects on the emotional burden of repeatedly having to explain and justify aspects of herself and her experiences to others. Her account suggests that the group provided a temporary respite from this labour, offering a space where understanding was assumed rather than negotiated. This experience resonates with broader research demonstrating how marginalised individuals are often required to justify, explain, or legitimise aspects of their identities and mental health experiences within everyday social interactions (Kaprinis & Charalampakis, 2025). Thus, the group and by extension, the programme, provided important protection from the pressures of having to mask aspects of their identities and experiences. Referring to themself as “broken,” Nicola frames the group as an important source of safety and belonging. While this language suggests a degree of self-denigration, it highlights the significance of finding a space in which vulnerability could be expressed without fear of judgement. For many in the group, becoming part of a collective with shared identities and experience is an important counter to the everyday heterosexism and stigma that they experience. As such, the programme offered connection, validation and safety from the emotional isolation many describe feeling outside of this context.

For many in the group some of the pressures described were linked to sexual and gendered identities under heteronormativity and gender norms that often ‘demand’ explanation:

*And that, you know, it gets tiring. It gets it’s exhausting, and especially when you’re like, OK, what questions are you are you going to ask that are inappropriate to ask but you don’t know. So I have to school you through that. Like there’s so many things of that. Meanwhile in this environment it was like you could go. Oh, I’m the whole I use they/them I’m bisexual and I’m gender fluid and they go cool.*

*(Yasmin)*

In this excerpt, Yasmin describes the emotional labour often required under heteronormativity as ‘*exhausting*’. Yasmin makes explicit reference to the identity-related questions she receives as examples of this, for someone who identifies outside of the gender and monosexual binary. This becomes more evident when she contrasts this with the programme context, where such emotional labour is not required. In turn, this underscores the protective role that community-based LGBT+ interventions offer when developed from, within and for the community.

For many participants, this was their first time performing comedy. The counsellor and facilitators were therefore essential in creating a safe and supportive environment that allowed participants to explore comedy in connection with their lived experiences and identities. Kelly emphasises this in the below excerpt:

*But for me, one of the really nice things was not just the security of knowing that [the counsellor] was there, but actually seeing the support that he was giving to other people because. There were people in the room that actively needed his support and he gave it to them. And whilst I don’t know what was discussed or what was going on, I could see it happening and that was a really, really positively impactful thing.*

*(Kelly)*

Kelly describes the support provided by the counsellor as an integral and ‘*impactful*’ component of the process, emphasising the responsive nature of this support. In this excerpt, Kelly highlights this as providing important foundation (‘security’) from which participants could engage while preserving a sense of safety, a feeling often absent in other contexts. The significance of this foundation for Kelly and the other participants can be better understood when situated against the backdrop of heteronormativity. Kelly’s account suggests that the group provided a rare environment in which participants did not need to monitor how they presented themselves or anticipate negative reactions from others. The ability to temporarily relinquish what is often referred to as hyper-vigilance appeared to facilitate feelings of comfort. Such experiences are particularly salient for minoritised individuals, who are often required to maintain heightened awareness of how their identities may be perceived and evaluated by others (Rostosky et al., 2022). As such, this support enabled participants to develop the necessary sense of safety and trust that allowed them to fully engage with and experience the benefits of comedy.

### 3.4. Comedy as Catharsis

Throughout the focus group discussions, participants described the positive impact comedy had on their mental health, highlighting the therapeutic potential of comedy for improving wellbeing. To capture the different ways these benefits were experienced, this theme comprised two interrelated sub-themes. The first sub-theme: Performing (queer) resilience, highlights the benefits of comedy as a tool for managing negative experiences, fostering new ways of coping with negative emotions and promoting greater emotional resilience. The second sub-theme: Taking the mic and owning the moment, highlights the empowering benefits participants experienced across different aspects of their lives, including improvements in confidence, self-esteem, and connectedness as a result of the programme.

### 3.5. Performing (Queer) Resilience

In reflecting on the factors that influenced their participation in the comedy programme, most referenced either past or ongoing mental health struggles as a driving force in this decision. Participants recognised the mental health benefits comedy offers, especially when done alongside other queer people. For everyone taking part, the specific offering of comedy *for* those within the LGBTQ+ community was central to their decision making.

*So yeah, for me it was the reason I signed up is I like comedy and I wanted to do it with queer people and also I was really intrigued to see the mental health [inaudible] because, I found when I first started trying to do comedy, it was really beneficial for my mental health and I will say it was exponentially more beneficial for my mental health doing it with a bunch of queer folk, so that’s good. So yeah, it was the linking of two things that are important to me improving mental health and improving my comedy skills.*

*(Kelly)*

For Kelly, it was specifically the *pairing* of comedy and the LGBTQteehee group that influenced her decision to take part. Having already experienced the mental health benefits that come from comedy, she recognised the additional benefits this offered within community and a programme that explicitly centres inclusivity. These benefits on mental health were echoed by all participants. For some, this served as an important way to help them deal with more acute periods of struggle, in addition to maintaining mental wellbeing more generally.

For Nicola, comedy has a therapeutic role in helping with her wellbeing, as stated in the following excerpt:

*And I just remember thinking particularly because it had the comedy and the mental health and like I say, it was always my coping mechanism. And I’ve always really struggled to talk about my mental health, even when I have been out of a crisis when, you know, it has been better. I’ve always struggled to talk about it and I still do to some extent, but being around other people that have mental health issues is easier because you don’t have to explain everything.*

*(Nicola)*

Here, Nicola shares the difficulties she experiences discussing her personal mental health struggles and how comedy helps her manage these struggles. Her account underscores the specific value of being able to do this alongside others with shared experiences, where there is no obligation to justify or explain. This illustrates the way in which comedy can be used as a means of enhancing resistance when experiencing mental health struggles.

The benefits of comedy in helping individuals process, manage and regulate mental health struggles was emphasised throughout discussions.

*I was having problems with mental health, still am, and I’ve always known that like being able to like joke with people like not even just pretend it’s not happening, but to actually do good jokes for people, that’s awesome. You know, even about it, you know just doing it with my friends and stuff. So for me, there definitely was an element of, oh, this will be fun and therefore that’s good for my mental health.*

*(Nazeem)*

Having shared his mental health struggles, Nazeem refers to the positive impact comedy has on his mental wellbeing. In recognising the role comedy plays as a positive coping mechanism, Nazeem explicitly describes comedy as a practice that supports mental health. Referred to here as doing ‘*good jokes*’, comedy, with its expressive qualities, this functions as a means of helping manage negative emotions. However, he identifies that there are continued struggles with his mental health, which is important to acknowledge, as the programme cannot be seen as a panacea to mental health. Instead it serves as one of many pathways to good mental wellbeing and resilience. Yet, similarly to others in the group, Nazeem describes this as a way processing emotions rather than a way of denying or avoiding them (‘*not just pretend it’s not happening*’). This speaks to the important benefits comedy plays around emotional processing and regulation, alongside helping foster resilience and psychological wellbeing. This is supported in the following extract where Yasmin describes the way she feels able draw upon her more traumatic experiences within her comedy, when doing so as part of a safe and validating space.

*So I don’t have to say, oh yeah, I’m fine and nothing’s ever bad’s happened. And you could go, hey, yeah I’m I’m thinking about making a joke about this really traumatic thing that happened to me. Can you guys help me make it funny? And it’s like, there was some shorthand that we could get to know each other so much better than like going into any like basically any other group.*

*(Yasmin)*

Yasmin reflects on how the supportive nature of the group made her feel more able to share the challenging aspects of her experiences, which in turn enabled her to form deeper connections with the group. We see here that comedy has an important relational and connective function, enabling people to get closer to others. This is further bolstered through how comedy can be used to work through difficulties or past negative experiences. The comfort to share these experiences speaks to the empowering role of comedy for therapeutic means, along with importance of the group in creating the necessary psychological safety.

Importantly, participants consistently identified the queer-specific nature of the programme as both a motivation for participation and a key source of its perceived benefit. Whilst resilience was developed through individual and community processes, these processes were grounded in shared experiences of queerness, mutual recognition, and identity affirmation. As such, the findings suggest not simply the development of community resilience in a generic sense, but resilience that was enacted through and shaped by queer identities and queer social connection.

As such, these accounts highlight the important role that community centred programmes offer in helping individuals process stressors and enhance emotional resilience when given the space to do so without fear of stigma or ridicule. This is vital when considering the chronic and negative effects of minority stress and the way this can often increase feelings of isolation and exacerbate mental health symptoms (Jaspal et al., 2023). The ability for comedy to counter minority stress further highlights the unique mental health benefits that community-based comedy interventions offer LGBTIQ+ individuals.

### 3.6. Taking the Mic and Owning the Moment

The positive and transformative nature of the programme was a prominent theme throughout participants accounts, and was evident on several different personal, social and professional levels. Participants emphasised the many positive effects the programme had on them and their everyday lives. Most notably, they shared changes in the way they respond and navigate mental health struggles, noting shifts from more avoidant coping strategies towards more active strategies. This is highlighted below where Kelly articulates positive changes and the increased comfort she experienced around emotional engagement as a result of the programme.

*But like I would, that’s normally what would be my approach. I would deflect and just say I’m amazing where actually inside I was the going I feel truly awful, whereas actually. I openly said it, which I think has been a big bit of growth for me. It’s a habit I would like to maintain.*

*(Kelly)*

Kelly describes having previously used deflection as a coping mechanism when experiencing negative emotions and having recognised this during the programme. Recognising that deflection did not help to manage negative emotions or ease distress represents significant progress for Kelly where this is framed as (‘*big bit of growth for me*’). As highlighted, more adaptive ways of coping are something that can be implemented because of greater emotional awareness and strategies learnt through this experience (‘*habit*’).

Like Kelly, Yasmin describes having experienced significant personal growth since participating in the programme and shared how she noticed the benefits in other contexts.

*But the difference is really stark. Like to the point where like people at work have noticed that I’ve, this phrase has haunted me since school, But I’ve come out of my shell even though I’ve been working there for four years. Like, you know, the difference between then and now and like they feel like I’m actually engaging more and like having jokes people more I can talk to people easier actually like interacting better.*

*(Yasmin)*

In describing the changes she has experienced resulting from the programmes as significant (‘*stark*’), Yasmin refers to observations by her colleagues who have noticed her increased confidence and engagement. She contrasts this with how she felt and was perceived prior to this, noting it as a more recent development having worked in the same workplace for a long period of time before the programme.

The propensity of the programme to help enhance personal and social growth was something that all participants noticed in themselves and in one another. While participants emphasised the role of comedy in supporting personal development, it was the importance of doing this within community that many found particularly transformative. We can see this most acutely in the following excerpt by Athena:

*I think we’ve all gained a new found confidence. It’s a confidence we feel I think. And it’s like gaining strength from each other from that group. It’s like stepping away from the group and you’re in the world. But you feel the support of that group, and it’s given us all confidence to be a bit more. Yeah. Say it. You know, we are all valid and all important. And comedy, I think, has brought us all together. But sort of given us a lot more as well, it’s made us all feel quite important, you know. It’s each other that’s done that I think within the group within the set up, given each other, the support to be themselves and you’ve practised in the group being yourself and then taking it out into the world, isn’t it? I think, so It’s built over into real life, which is fabulous.*

*(Athena)*

Drawing on others’ accounts of the benefits of the programme and community, Athena summarises what she feels are shared group benefits. This is characterised as increases in personal growth, confidence and self-worth, along with growth in relationships as a direct result of the support provided by the group. The explicit reference to increased confidence and self-worth, fostered through experiences of validation within the group (“*being yourself*”) and recognised as a collectively benefit (“we are all valid and all important”), highlights the central role community plays in fostering individual and shared outcomes. This further illustrates the emotional and identity-related benefits of community-based programmes for marginalised individuals.

## 4. Discussion

This study explored LGBTQ+ participants’ experiences of “LGBTQTeehee!”, a 10-week stand-up comedy intervention, and how they made sense of its perceived impact on their wellbeing and resilience. Participants described the programme as providing opportunities for community connection, emotional expression, and personal growth, with many reflecting on the importance of engaging in comedy within an affirming queer environment. Reflexive thematic analysis identified two central processes underpinning the programme’s perceived efficacy: the development of a supportive LGBTQteehee group, and empowerment through the creation and performance of comedic narratives. Importantly, these findings suggest that the benefits of the intervention extend beyond humour itself, instead reflecting the interplay between social connection and identity-affirming self-expression as key mechanisms through which wellbeing is supported.

The development of social connection and community building is a foundational component of social identity approaches to health and wellbeing (Jetten et al., 2012, 2017; Peters et al., 2024; Steffens et al., 2021). The present findings strongly align with this perspective, reinforcing the importance of group-based belonging as a mechanism for supporting mental health. However, the explicitly LGBTQ+ nature of the intervention added an important additional layer, with participants emphasising a shared sense of safety and tacit understanding that was central to their experience (McKenzie et al., 2024). This is particularly significant given that queer spaces remain under threat, and opportunities for meaningful cultural engagement, especially those that centre marginalised identities, are not equally accessible (Brook et al., 2025).

Notably, participants described connection not only through shared identity, but through diversity within the group. While “LGBTQteehee!” brought individuals together under a broad queer umbrella, participants often differed across gender identities, sexualities, and lived experiences, united instead by a shared interest in comedy. This challenges traditional interpretations of in-group processes that prioritise similarity and assimilation, suggesting instead that shared purpose may be sufficient to enhance cohesion (Hornsey & Jetten, 2004). In this sense, the findings highlight the value of diverse queer spaces that facilitate organic connections between individuals who may not otherwise interact, expanding the scope of what constitutes meaningful group belonging. This is particularly important given recent scholarship suggesting that community space alone is insufficient. Instead, resilience emerges through active processes of space making that transform shared experiences of adversity into collective empowerment and action (Tate et al., 2025). Without such processes, communities may risk becoming sites of trauma bonding rather than growth and resistance. Within this context, “LGBTQteehee!” demonstrates the potential of comedy-based interventions to facilitate resilience as an active and agentic process rather than a passive one (Bartlett et al., 2022; Lockett et al., 2023).

Psychological safety emerged as a vital underpinning of these processes. As a queer-led initiative, the programme appeared to cultivate an immediate sense of sharedness, characterised less by formal facilitation and more by mutual recognition and understanding. Participants described relief from the fatigue associated with navigating heteronormative environments, where identity often requires explanation or justification (Bry et al., 2017; Jaspal, 2025). Within this space, shared lived experience functioned as a form of tacit knowledge, enabling participants to express themselves without the need for extensive self-explanation, and allowing support to be offered intuitively.

However, these findings also raise important considerations for future implementation. The success of the programme was closely tied to its queer-led design, including the presence of facilitators and mental health support that were culturally competent and affirming. As such, future interventions should prioritise these elements, ensuring that psychological safety is actively cultivated through appropriate facilitation, embedded support structures, and careful risk management (Iacono, 2019).

The use of lived experience as material for stand-up comedy is well established within the art form (Barnes, 2025; Double, 2017; Lindfors, 2019). However, narratives surrounding mental health and minority stress are often mediated through structured and bounded contexts such as therapeutic settings, where expression, while potentially candid, is shaped by concerns around safety, risk, and the limits of existing frameworks for understanding trauma (Brown, 2024). In contrast, participants in the present study described the act of transforming lived experience into stand-up comedy as profoundly beneficial. While this form of performance may not be suitable for all individuals, its emphasis on solo performance brings to light a particularly striking dimension of resilience.

For many participants, humour was already an established coping mechanism; however, the programme appeared to extend this from an interpersonal or internal strategy into a performative and transformative process (Camfield & Beharry, 2025). In doing so, participants were not simply managing difficult experiences but actively reshaping them—reframing narratives of minority stress and trauma into forms that were agentic, controlled, and, crucially, empowering. This process of narrative reclamation represents a distinctive contribution of the intervention, suggesting that stand-up comedy may offer a unique mechanism for meaning-making that warrants further exploration.

Importantly, this process did not occur in isolation. While stand-up comedy is often characterised by individualism and competitive progression (Sedgwick, 2024), the structure of the programme developed a sense of collective efficacy (Häusser et al., 2020). Although the final performance was delivered individually, the developmental journey was shared, with participants supporting one another in crafting and refining their material. The culmination of the programme, in which all participants performed their sets, was therefore experienced not only as individual achievement, but as a collective success.

This interplay between individual performance and collective support is particularly significant within a queer context (Techakesari et al., 2017). At an individual level, participants described using comedy to reinterpret difficult experiences and engage more openly with their emotions (i.e., reattribution). At a group level, participants emphasised the value of shared understanding, validation, and mutual support, which acted as a form of “shorthand” that enabled difficult experiences to be transformed into comedy without extensive explanation—developing a sense of collective self-efficacy (Häusser et al., 2020; Hornsey & Jetten, 2004). In this sense, the intervention functioned not only as a creative outlet, but as a space in which personal reflection and community connection operated in tandem. For LGBTQ+ individuals, whose experiences are often shaped by minority stress and marginalisation, the act of “standing up” to publicly reclaim and reinterpret personal narratives carries both symbolic and psychological weight. In this sense, the intervention functioned not only as a creative outlet, but as a form of collective empowerment, enabling participants to transform past adversity into a resource for present resilience and future-oriented identity construction.

### Implications and Limitations

Whilst “LGBTQteehee!” remains a novel intervention in the early stages of development, several tentative implications emerge from the present findings. First, the study adds to a growing body of literature suggesting that arts-based interventions may represent valuable avenues for supporting mental wellbeing and, by extension, public health. Although the present findings cannot speak to efficacy, participants consistently described the programme as a meaningful space for self-expression, connection, and reflection, lending support to wider calls for greater investment in creative and community-based approaches to wellbeing.

Of particular interest is the combination of comedy and queerness. Whilst humour has long been discussed as a coping strategy within LGBTQ+ communities, with debates around its effectiveness, participants’ accounts suggest that the benefits of the programme were not derived from comedy alone. Rather, the programme appeared to provide a structured and affirming queer space in which humour could be used collectively to explore difficult experiences and enhance connection. This highlights the potential importance of pairing creative practice with meaningful opportunities for identity affirmation and community building in an agentic and meaningful way.

These findings may also have broader relevance given ongoing concerns regarding the decline of dedicated LGBTQ+ community spaces (Doan & Higgins, 2011; Matsick et al., 2024). Moreover, the programme allowed for connecting with other queer individuals outside of alcohol-centric environments, suggesting that arts-based programmes may offer alternative routes to community engagement and belonging (Hunt et al., 2019). Whilst speculative, this raises the possibility that interventions such as “LGBTQteehee!” could contribute not only to individual wellbeing but also to the strengthening of community connections and opportunities for collective resilience.

There are, of course, several limitations to the present study. First, while qualitative research does not seek statistical generalisability, further work is required to explore the transferability of these findings across different contexts and populations. Future research may benefit from mixed methods designs (including quantitative analyses) that incorporate longitudinal measures of mental wellbeing and resilience to examine changes over time. Second, as a novel intervention, the study involved a relatively small and self-selecting participant group. While this enabled in-depth exploration of participant experiences, it necessarily limits the breadth of perspectives captured. At this juncture, it is also pertinent to reflect on the participant accounts that were not captured. Although all nine participants successfully completed the programme, three did not participate in the follow-up data collection. As such, the present findings are based on the experiences of those who elected to share their reflections after the intervention. Whilst there is no indication that non-participation reflected dissatisfaction with the programme, these missing accounts may represent experiences, perspectives, or tensions that were not captured within the analysis. Consequently, the findings should be interpreted with appropriate caution when considering the feasibility and acceptability of the intervention more broadly.

A further limitation concerns the ethnic composition of the sample. Most participants identified as White, limiting our ability to explore how ethnicity and race may intersect with LGBTQ+ identities in shaping experiences of the intervention. Replication with larger and more diverse samples would be valuable in assessing the consistency and scalability of the observed benefits. Finally, data were collected shortly after the completion of the programme, meaning that longer-term impacts remain unclear. Future evaluations should incorporate follow-up periods (e.g., 6–12 months post-intervention) to assess the sustainability of outcomes. Where feasible, the inclusion of comparison or control groups would also strengthen causal inferences regarding the effectiveness of comedy-based interventions.

## 5. Conclusions

In conclusion, “LGBTQteehee!” was experienced as an effective programme for supporting and enhancing the mental wellbeing and resilience of LGBTQ+ individuals. These findings contribute to growing calls for arts-based approaches to be taken seriously within public health strategies, particularly as accessible and community-driven forms of support. Central to the success of the programme was the creation of a shared queer space underpinned by psychological safety, which enabled participants to engage authentically and form meaningful connections.

While transforming lived experience, and, in some cases, minority stress and trauma, into stand-up comedy may not be suitable for all individuals, for those involved it functioned as a powerful form of narrative reclamation. Participants were able to harness humour, a common coping mechanism within marginalised communities, and develop it into an expressive and empowering performance practice. In doing so, the intervention not only facilitated individual wellbeing, but also fostered collective connection and resilience, highlighting the unique potential of community-based comedy as a tool for supporting LGBTQ+ mental health.

## Figures and Tables

**Table 1:** Pseudonyms and data collection format.

Pseudonym	Focus Group	1-2-1 Interview
Kelly	✓	
Athena	✓	
Nicola	✓	✓
Nazeem	✓	✓
Yasmin	✓	✓
Sameer		✓
Sue		
Nova		
Eliza		

## Data Availability

Due to the sensitive nature of qualitative data, anonymized data can only be requested from the corresponding author with a reasonable request.
